# A Three-Dimensional Skeletal Reconstruction of the Stem Amniote *Orobates pabsti* (Diadectidae): Analyses of Body Mass, Centre of Mass Position, and Joint Mobility

**DOI:** 10.1371/journal.pone.0137284

**Published:** 2015-09-10

**Authors:** John A. Nyakatura, Vivian R. Allen, Jonas Lauströer, Amir Andikfar, Marek Danczak, Hans-Jürgen Ullrich, Werner Hufenbach, Thomas Martens, Martin S. Fischer

**Affiliations:** 1 AG Morphologie und Formengeschichte, Bild Wissen Gestaltung–ein interdisziplinäres Labor & Institut für Biologie, Humboldt-Universität, Berlin, Germany; 2 Institut für Spezielle Zoologie und Evolutionsbiologie mit Phyletischem Museum, Friedrich-Schiller-University, Jena, Germany; 3 Structure and Motion Laboratory, Royal Veterinary College, North Mymms, United Kingdom; 4 Das Department Design, Hochschule für Angewandte Wissenschaft, Hamburg, Germany; 5 Institut für Leichtbau und Kunststofftechnik, Technical University, Dresden, Germany; 6 Museum der Natur, Stiftung Schloss Friedenstein, Gotha, Germany; University of Oxford, UNITED KINGDOM

## Abstract

*Orobates pabsti*, a basal diadectid from the lower Permian, is a key fossil for the understanding of early amniote evolution. Quantitative analysis of anatomical information suffers from fragmentation of fossil bones, plastic deformation due to diagenetic processes and fragile preservation within surrounding rock matrix, preventing further biomechanical investigation. Here we describe the steps taken to digitally reconstruct MNG 10181, the holotype specimen of *Orobates pabsti*, and subsequently use the digital reconstruction to assess body mass, position of the centre of mass in individual segments as well as the whole animal, and study joint mobility in the shoulder and hip joints. The shape of most fossil bone fragments could be recovered from micro-focus computed tomography scans. This also revealed structures that were hitherto hidden within the rock matrix. However, parts of the axial skeleton had to be modelled using relevant isolated bones from the same locality as templates. Based on the digital fossil, mass of MNG 10181 was estimated using a model of body shape that was varied within a plausible range to account for uncertainties of the dimension. In the mean estimate model the specimen had an estimated mass of circa 4 kg. Varying of the mass distribution amongst body segments further revealed that *Orobates* carried most of its weight on the hind limbs. Mostly unrestricted joint morphology further suggested that MNG 10181 was able to effectively generate propulsion with the pelvic limbs. The digital reconstruction is made available for future biomechanical studies.

## Introduction

The discovery of Devonian, Carboniferous and lower Permian fossils has provided insight into the early evolution of tetrapods—especially into the evolution of limbs and early diversification within the new terrestrial habitat (e.g., [1]). The understanding of the functional morphology of transitional forms, which are not represented by adequate modern analogues, is unfortunately often hampered by taphonomic and diagenetic effects—incomplete, disarticulated, crushed, and distorted fossils. Moreover, fossils of this timespan often are fragile and the surrounding matrix cannot completely be removed without risking damage to the specimen, and so the valuable holotype and paratype specimens understandably are often not accessible for such extensive and/or destructive analyses. Given that digital reconstructions offer diverse possibilities for further analyses (e.g., visualizing internal structures, virtual endocasts, etc.) researchers increasingly rely on non-invasive imaging and three-dimensional (3D) measurement techniques for computer-aided analysis of skeletons to study the transition of vertebrates to land (e.g., [2–6]). Digital models have been used to estimate biomechanical parameters such as joint range of motion (e.g., [4,7,8]), mass parameters (e.g., [9,10]) or are used as the basis for musculoskeletal simulations (e.g., [11,12]).

The evolutionary transition of early tetrapods to fully terrestrial habitats was completed with the appearance of amniotes, which do not possess aquatic larvae, but develop directly within an egg equipped with an internal fluid and food reservoir (e.g. [13]). Amniotes are a successful clade and represent roughly 75% of modern tetrapod species, but modern amniotes are highly derived from their last common ancestor. The mostly Permian diadectids are widely considered to represent the fossil sister group to modern amniotes and therefore potentially share many characteristics with the first amniotes (e.g., [14–16]).

*Orobates pabsti* (Holotype MNG 10181, [17]) is one of the most basal diadectids, from the lower Permian of Germany (Bromacker quarry, Tambach Formation, Tambach-Dietharz, Thuringia). Because of its phylogenetic position, this nearly complete, articulated, comparatively well-preserved specimen can be considered a key fossil. Additionally, fossil trackways from the ichnospecies *Ichnotherium sphaerodactylum* of the same locality were unequivocally produced by *Orobates pabsti* rendering this combination of body fossil and fossil trackways the oldest known track-trackmaker association and offering direct evidence of its locomotor behaviour [18]. In sum, MNG 10181 is an ideal candidate for an attempt to reconstruct the locomotor characteristics of a stem amniote close to the anamniote/amniote transition. However, like most fossils this specimen suffers from the taphonomic and diagenetic problems mentioned above, and so some degree of reconstruction is a necessary precursor to detailed morpho-functional and biomechanical analyses. In this paper we describe our workflow for the digital three-dimensional (3D) reconstruction of MNG 10181 to be used in further biomechanical analyses. As a first application of the new 3D reconstruction we estimated mass properties of individual segments and the whole body and analysed range of mobility in the shoulder and hip joints.

### Taphonomy of MNG 10181

The fossils of the Bromacker quarry appear in a sequence together with large sediment filled burrows and typical burrow structures (*Megatambichnus* sp.) seemingly produced by the diadectids [19,20]. One of the reasons this location produced relatively well articulated and complete tetrapod fossils might be that the diadectids were inaccessible for predators after death when trapped and buried in an event of burrow collapse. Moreover, there is no evidence of transport of parts of the animals after death [21]. Typically, the soft tissues decay and are replaced by clay or calcite, Hollow parts of the skeleton (e.g., the skull) may partly collapse or be filled with sediment.

**Fig 1:**
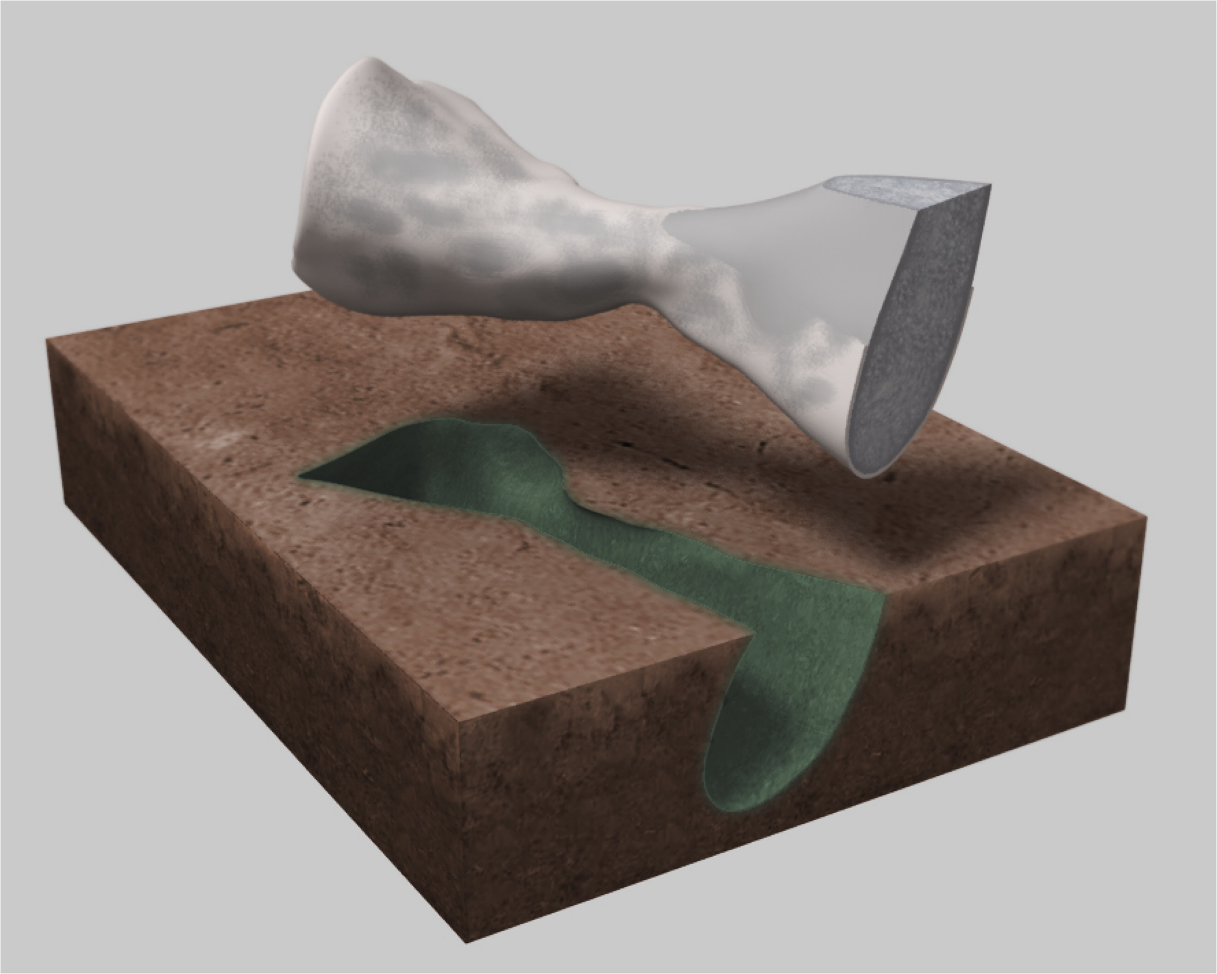
Schematic illustration of the state of fossil vertebrate material due to taphonomic processes. The bone was fossilized into aragonite (grey-blue) and is enclosed in a thin layer of crystalline calcite (white). The fine-grained alkaline sediments (siltstone) around the bones usually turned grey-green in the process.

The dead animals, including MNG 10181, were covered mainly by fine-grained sediments with alkaline chemical composition. After decay of the soft tissues, the organism was affected by chemical changes that involved the transport of chemicals in solution within the buried sediment. This lead to significant chemical substitution around the bones and the bones were mostly enclosed in a thin crystalline calcite layer (Fig 1). The bone itself was fossilized into white or blue-grey calcite and/or aragonite. After these chemical processes, the fossilized bones still exhibit most parts of their original structures.

**Fig 2:**
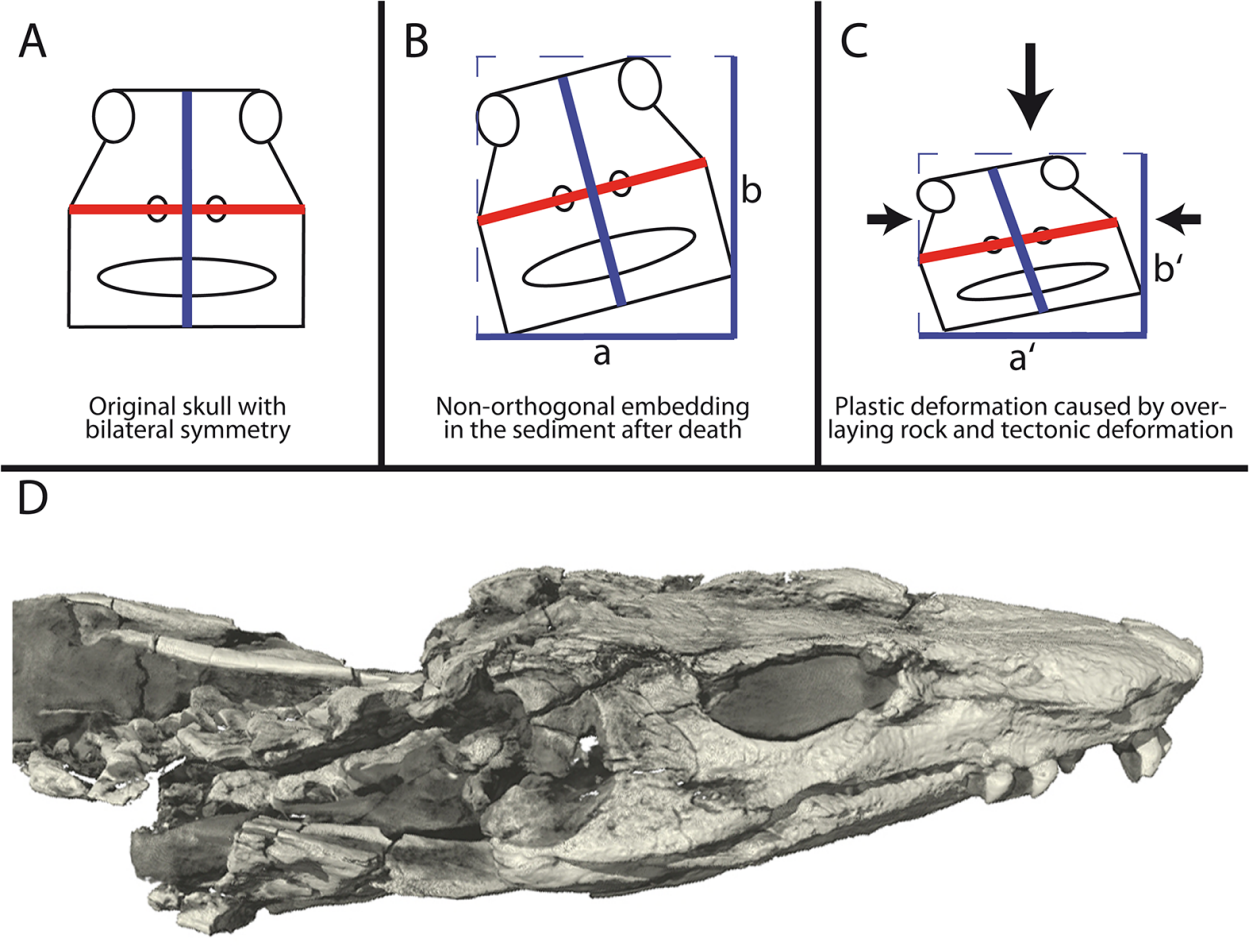
Diagenetic distortion of the bone bearing strata at the Bromacker quarry result in plastic deformation of the fossils. A: The original skull was bilateral symmetric with perpendicular rostro-caudal (not shown), latero-lateral (red), and dorso-ventral (blue) axes. B: Preservation is non-orthogonal to main directions of diagenetic distortion resulting in complex plastic deformation of a fossil. C: The main directions of diagenetic distortion were vertical compaction (large arrow) and, to a significantly lesser extent, horizontal tectonic deformations (smaller arrows). D: A volume render of the skull of MNG 10181 to illustrate the extent of plastic deformation, fractures of bones, and the high resolution of the CT scan data.

The Tambach Formation was overlaid by different rock formations of up to 2000 m in thickness over a time span of approx. 295 million years leading to significant vertical compaction of the fossil bearing stratum [22]. Depending on the orientation of the fossil (isolated bones or complete articulated skeletons like MNG 10181) in the sediment, the vertical compaction resulted in one-directional plastic deformation (Fig 2). Moreover, rupture fissures filled with calcite suggest an additional horizontal deformation of significantly lower effect than the aforementioned vertical compaction [23,24]. Importantly for the purpose of this study, diagenetic processes likely affected the whole bone bearing stratum in the same way leading to approximately uniform plastic deformation of the whole articulated holotype skeleton of *Orobates pabsti* (MNG 10181).

## Materials and Methods

### Computed tomography of MNG 10181

No permits were required for the described study, which complied with all relevant regulations. All specimens (MNG 10181, MNG 8760, MNG 8980, and MNG 8966) used for this study are housed at the Museum der Natur, Gotha. The preservation of the holotype specimen MNG 10181 within rock matrix made non-destructive imaging necessary to acquire shape information of fossilized bone fragments. The considerable size of the specimen (total dimensions with surrounding rock matrix approx. 1.0 m x 0.8 m x 0.35 m; approx. 35 kg) and the related substantial absorption of x-ray photons posed practical problems, because most micro-focus computed tomography (CT) scanners are not equipped to handle objects of this size (i.e., the penetration depth is limited). We used a large micro-focus CT scanner (v|tome|x L450, GE phoenix|x-ray systems, Wunstorf, Germany) at the Institut für Leichtbau und Kunststofftechnik at the Technical University in Dresden, Germany. The scanner can handle even larger objects of up to 200 kg, while offering two separate x-ray tubes. The ‘smaller’ tube (maximal acceleration voltage 300 kV acceleration voltage; maximal power 500 W) allowed highly detailed CT-scans (up to 62 μm resolution) of fossil material that was separate from the main block (see below). For the main block, the more powerful tube (maximal acceleration voltage 450 kV; maximal power 4.5 kW) was used. The detector had a field of view of 400 x 400 mm (2024 x 2024 pixels), at a resolution of 150 μm.

**Fig 3:**
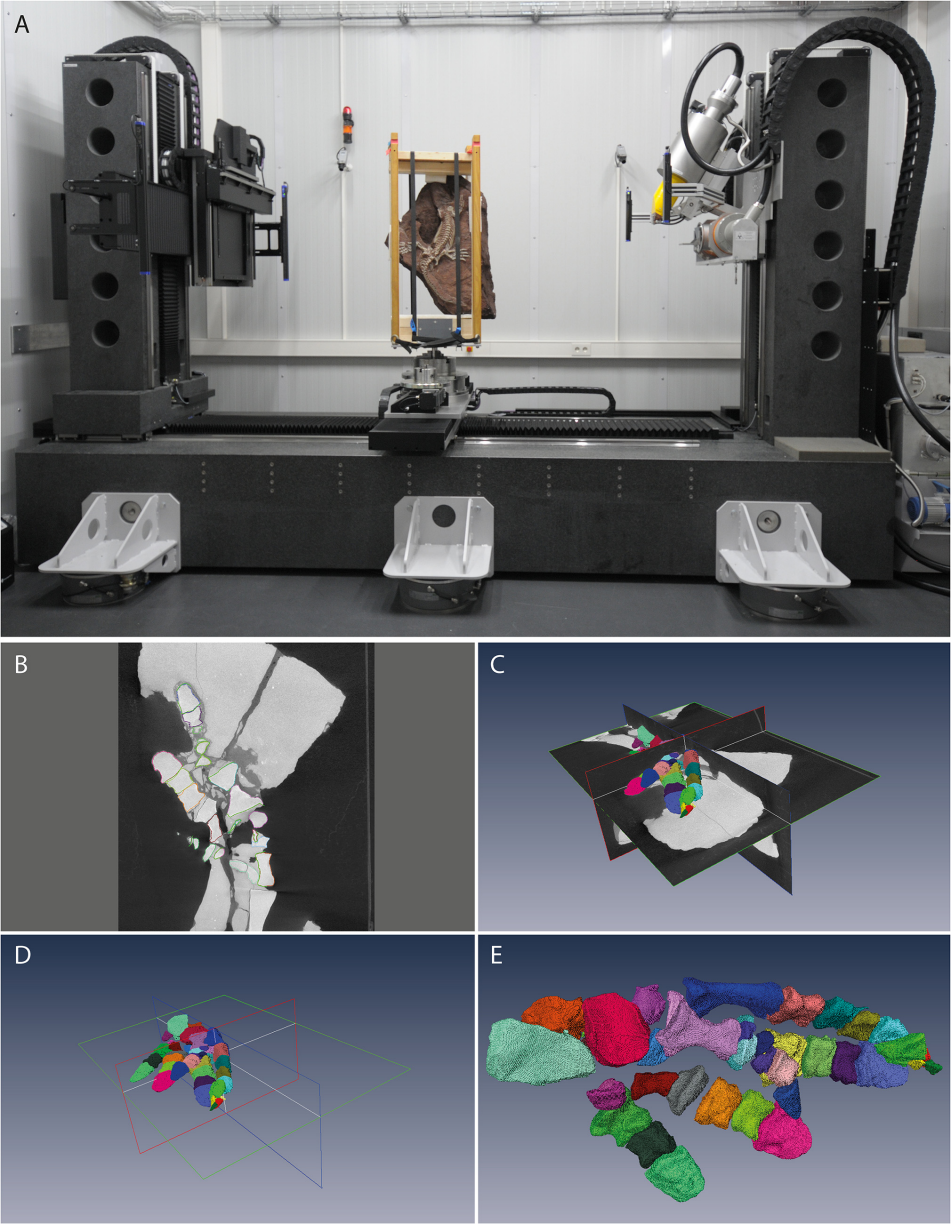
CT scanning of the holotype specimen of *Orobates pabsti* (MNG 10181) and segmentation of bones and bone fragments from CT image stacks in Amira (here left manus shown as an example). A: The main block of the specimen mounted within the v|tome|x L450, (GE phoenix|x-ray systems, Wunstorf, Germany) at the Institut für Leichtbau und Kunststofftechnik in Dresden, Germany. B: Because grey level differences between fossil bone and surrounding matrix were minimal and heterogeneous, outlines of bones had to be traced by hand on individual images of the stack. C: Bone outlines were traced on as many images as necessary to yield the correct 3D bone shape from interpolation. D, E: Voxels assigned to a bone were extracted from the matrix and volumes subsequently surface rendered.

MNG 10181 was scanned within a custom-built radio-transparent fixture that allowed rotation of the specimen along the longest axis of the specimen and reduced unintended motion from repeated stopping/starting of rotation during the scanning procedure (Fig 3).

Highly detailed CT-scans were acquired for the skull, the cervical vertebrae, cervical ribs, the shoulder girdle, and the left forelimb (240 kV; 140 μA; resolution 75 μm; 2 h acquisition time; Figs 2 and 3). These parts of the skeleton were separated from the main block during preparation (cf. [17]). Micro-focus CT scans of lower resolution taken of the main fossil block were used to reconstruct the pelvis and the left hindlimb (450 kV; 1.5 mA; 150 μm; 8 h acquisition time). Before exporting for further analysis, the image stack was filtered to enhance the contrast between fossil bone and surrounding matrix using the CT system’s software package (datos|x 2.0, GE phoenix|x-ray systems, Wunstorf, Germany). Right limbs were simply mirrored at a later stage of the skeletal reconstruction (see below). Post cervical vertebrae and ribs could not be reconstructed from CT scans, because the x-rays could not penetrate the fossil block from sufficient angles. Therefore, these structures had to be modelled. However, we successfully scanned isolated fossil vertebrae (MNG 8980 and MNG 8966; 250 kV; 180 μA; 62μm; 1 h scanning) and used these as templates for the modelling of post cervical vertebrae. Importantly, we made sure that the modelling did not contradict the detailed description of the same material by Berman and colleagues [17]. Notably, the previous publication includes detailed descriptions of the size and shape of the neural spines, neural arches, and overall dimension of each vertebra [17]. We modelled the exact number of vertebrae of the holotype specimen MNG 10181 which appears to be complete, but acknowledge that the paratype (MNG 8980) apparently has a higher count of caudal vertebrae [17].

### Segmentation, repairing and modelling of bones

**Fig 4:**
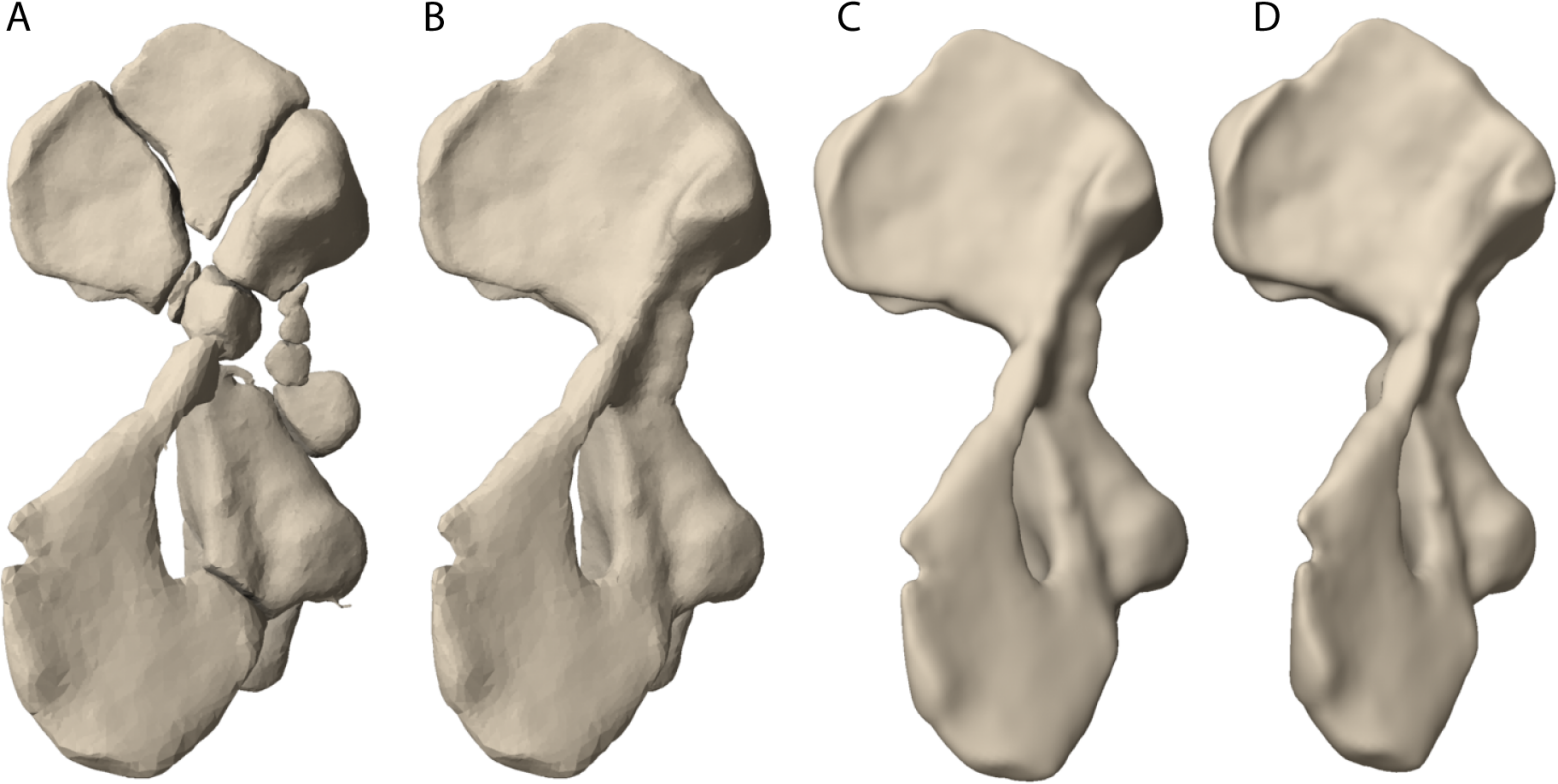
Workflow for the restauration of bone models here shown for the left humerus. A: bone models segmented from the CT data were fragmented and suffered from plastic deformation. B: after importing bone models into Maya, fractures were repaired using available modelling tools. This step resulted in ‘watertight’ bone models. C: the surface mesh was re-mashed in ZBrush to more parsimonious quad-meshes to drastically reduce polygons and hence file size. D: surface models were un-distorted as explained in the text (also see Fig 5).

As expected from the taphonomic and diagenetic alterations of the biological material, grey level differences on CT images between fossilized bone and surrounding matrix were very heterogeneous which meant that computer segmentation algorithms could not be used. Segmentation was done manually using the segmentation editor in Amira by tracing the outlines of bone fragments on individual images of the CT image stack (Fig 3). The segmentation editor of Amira facilitates interpolation of bone outlines between non-consecutive images of the stack. However, the interpolation often introduced errors. In these cases, we re-traced the outline in interpolated images until bone fragments were correctly digitized in all three dimensions (Fig 3). Subsequently, the surrounding matrix could be removed and digital bone fragments were modelled as 3D rendered surfaces. At first, all bone fragments were treated as individual objects. Bone fragment surfaces and information of size and relative orientation were imported into Maya. Fragments belonging to the same bone were combined, cracks and fissures were repaired using the modelling tools of Maya and watertight bone surfaces were derived (Fig 4). The resulting, extremely high-detail meshes, were then reduced using the remesh tool in *Z*Brush™ (Pixologic™, Los Angeles, CA, USA).

### Correction for distortion and virtual mounting

**Fig 5:**
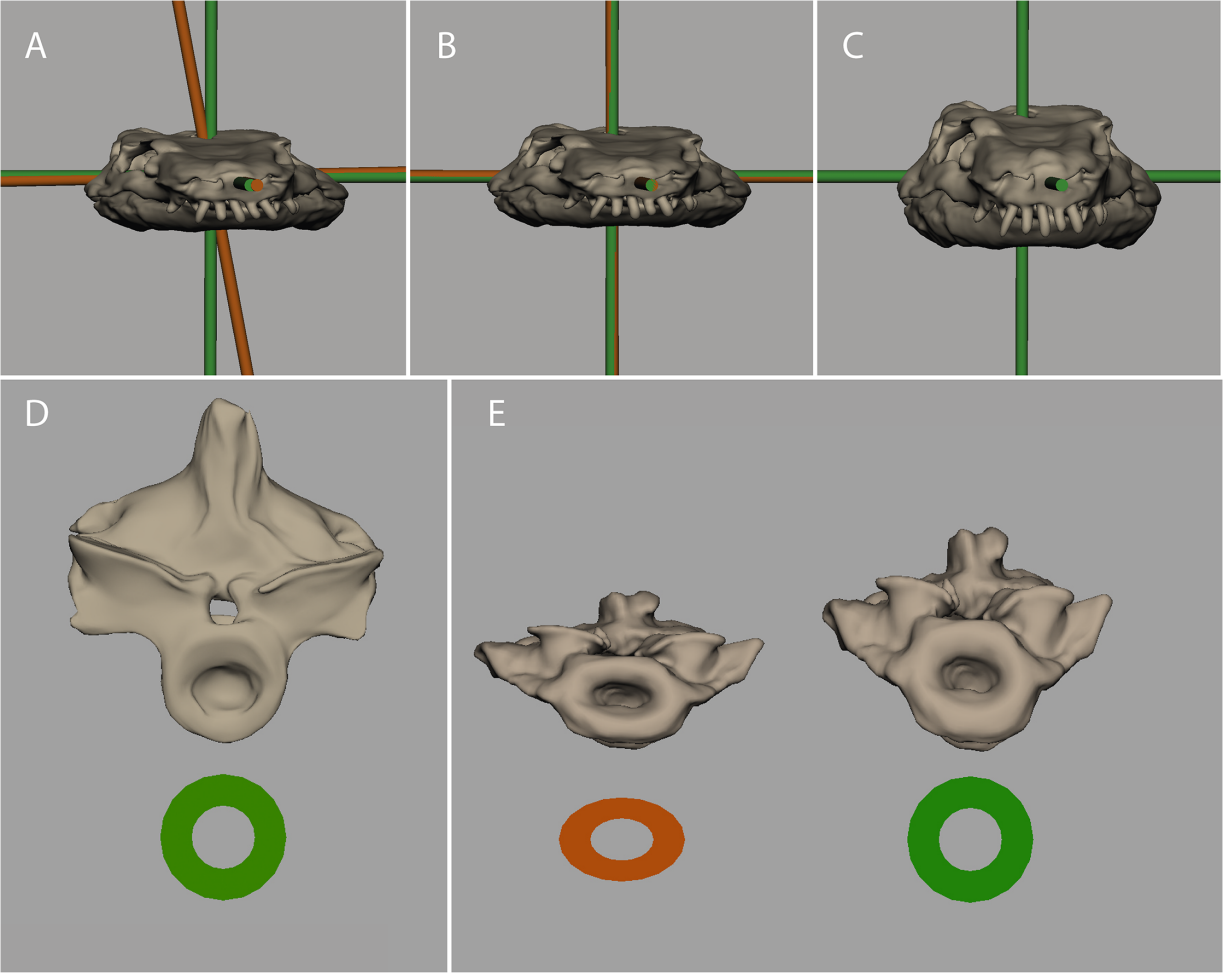
Using simple lattice deformers to undistort MNG 10181 in Maya. **A, B: Direct evidence was used to restore bilateral symmetry.** In (A) the original, distorted skull with rostro-caudal, latero-lateral, and dorso-ventral axes (bronze) is shown and distortion relative to the perfectly perpendicular green axes is obvious. By deforming a lattice containing the skull and the bronze axes so that the bronze axes match the green axes (B) bilateral symmetry is restored (rule 1). C, D, E: Circumstantial evidence used to correct for dorso-ventral flattening. In (D), the isolated vertebra MNG 8966 is not dorso-ventrally flattened due to preservation in flat orientation and has a round neural centrum. In (E), the fifth cervical vertebra of MNG 10181 displays dorso-ventrally flattened neural centrum (bronze) and was corrected until neural centrum had a round shape again (rule 2). Both rules were used to correct distortion for all CT derived bone models of the holotype specimen (C).

To correct for diagenetic distortion of the holotype specimen we made use of direct and circumstantial evidence. We acknowledge that in principle (and if studied close enough) such deformation will always be heterogeneous, but for the purpose of this study we assumed homogeneous deformation that affected the whole bone bearing stratum. First, we corrected shear deformation and restored bilateral symmetry of the skull. To this end, we assumed perfectly perpendicular latero-lateral, dorso-ventral and caudo-rostral axes (also see [25]). By fitting these axes into the distorted skull model the degree of distortion becomes evident (Fig 5). The non-perpendicular axes of the distorted skull were parented to the skull in Maya. The skull was then undistorted using lattice deformers until the axes were perpendicular again (rule 1).

Due to the orientation of MNG 10181 in the block most of the deformation occurred in the form of dorso-ventral flattening. We needed to rely on circumstantial evidence to correct the amount of flattening. Fortunately, the Bromacker quarry also produced an isolated vertebra (MNG 8966), which was preserved with its cranio-caudal axes tilted by 90° (i.e., the single vertebra lay flat and hence was distorted not along its dorso-ventral axis, but along its cranio-caudal axis). Because in the isolated vertebra specimen the centrum is almost perfectly round we assumed this to also be the case in the dorso-ventrally distorted vertebrae of MNG 10181 in upright preservation. Again using lattice deformers in Maya, we undistorted the vertebrae of MNG 10181 until their centra appeared round (rule 2). As a cross-check, we also undistorted the isolated vertebra along its cranio-caudal axes by the same rate to check whether the dimensions of the vertebrae in upright preservation were met. This was the case.

Finally, we fitted all distorted CT derived bone models of MNG 10181 onto an image of this specimen to account for the orientation of individual bones within the fossil block. Then we applied rules 1 and 2 to undistort the holotype specimen, i.e., we assumed that plastic deformation effected the whole bone bearing stratum in the same way (see above). We then added the modelled bones and mirrored the limbs.

### Body mass properties estimation

We estimated body mass and centre of mass (CoM) for MNG 10181 using a computer model of body shape based on the reconstructed skeleton. We reconstructed body shape in individual segments (cranial, cervical, abdominal, 2 pectoral limbs and 2 pelvic limbs, tail) using three-dimensional modelling software (Autodesk 3DSmax 2014), following a previously established method [26–28]. We estimated mass and CoM for individual segments using custom Matlab code (see S1 Code), based on their volume and standard density of 1000 kg m^-3^. We then calculated whole-body mass using Eq 1 (below) and whole-body CoM using Eq 2 (below).

$$M_{body}=\sum_{i=1}^{n}m_{i}$$ (1)

$$CoM_{\left[x,y,z\right]}=\frac{1}{M_{body}}\sum_{i=1}^{n}m_{i}r_{i\left[x,y,z\right]}$$ (2)

M_body_ is the total body mass, m_i_ is the mass of segment i and r_i_ is the distance from the system origin to the CoM of segment i (calculated as separate values for each set of x, y and z coordinates). The system origin was defined as a point midway between the hips in the reference pose.

We included a sensitivity analysis to account for uncertainty in the true body dimensions of MNG 10181. We varied radial dimensions for each segment between their initial skeleton-hugging values and 120% to simulate a ‘minimal’ (i.e., ‘initial’) and ‘maximal’ (120%) body outline (after [9,26–29]). To account for uncertainty of cross-section profile, we also scaled diagonal vertices of each octagonal outline hoop from their initial values (elliptical profile) to 120.7% (profile intermediate to ellipse and square) for the ‘maximal’ outlines and to 85.3% (profile intermediate to ellipse and diamond) for the ‘minimal’ outlines (after [30]).

Using equations one and two (above), we then analysed different combinations of ‘maximal’ and ‘minimal’ segments to represent the most cranial and caudal distributions of body mass, and the maximum and minimum total mass. This resulted in a range of plausible values for both CoM position and body mass.

### Mobility in shoulder and hip joints of MNG 10181

Mobility was assessed in the shoulder and hip joints by manipulating the digital humerus and femur models, respectively, until collision of these bones with the respective girdle element. We quantified maximal humeral and femoral protraction, maximal retraction, maximal abduction, maximal adduction and long axis rotation (LAR) using the same methodology as in a previous study on the hip of the green iguana [31]. Briefly, we used Maya joints to hierarchically combine the respective girdle with the humerus and femur, with the long bones being subordinated to their respective girdles. By implementing an anatomically defined joint coordinate system (ACS) into the approximated centre of rotation of the shoulder and hip, respectively, it was possible to measure the potential mobility directly within the software. The centres of rotation were found as the centre of a sphere fitted into the proximal joint surface of the humerus and femur, respectively. Each sphere was scaled and positioned to approximate the curvature of the joint surface. We acknowledge that modelling these joints essentially as ball and socket joints is an oversimplification, but the steps taken here allow reproducibility. Note that this method can only be used to determine the maximal mobility in terms of rotations around the perpendicular axes of the ACS of the specific joint and help to identify potential restrictions of mobility due to bony structures. The method ignores the confounding influence of soft tissues within and surrounding the joint, and the fact that *in vivo* movement during locomotion often utilizes just a fraction of the potential mobility (see discussion in [4,31,32]). We tested, however, the sensitivity to the assumed joint space between the bony surfaces of a joint by measuring the maximal mobility from zero joint space to 2.5 mm joint space with 0.5 mm increments. Moreover, this method ignores translatory motions in the joints, which are likely to have occurred given the elongate shape—especially of the glenoid.

Mobility was measured relative to a reference pose. We chose a non-physiological “sprawling” posture, with the humerus and femur oriented perpendicular to the sagittal plane and rotated about their long axes so that the medial and lateral epicondyles were in a horizontal plane. The *x*-axis of the right handed ACSs represented the long axis of the humerus and femur, respectively, and rotations around this axis were long axis rotations. The *y*-axis of the ACSs was pointing up and rotations around this axis were humeral and femoral pro- or retractions, respectively. The *z*-axis was pointing cranial and rotations around this axis were humeral and femoral ab- or adductions. Note that the orientation of these ACSs is different from the one at the system’s origin used for the estimation of CoM position, but is aligned with the bones in our reference pose and placed at the estimated joint centres of rotation.

## Results

**Fig 6:**
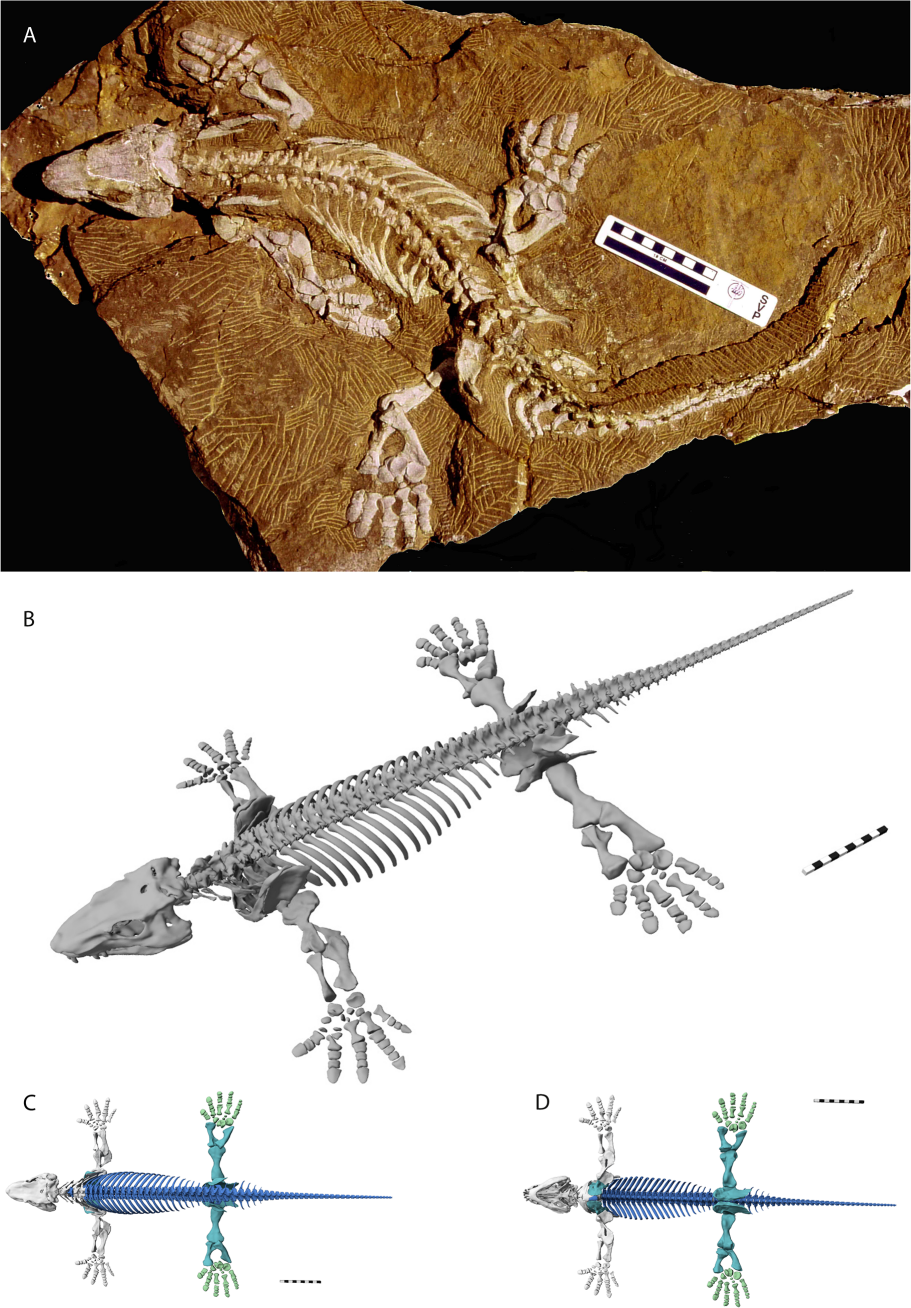
Digital reconstruction of *Orobates pabsti* (MNG 10181). A: Holotype specimen of *Orobates pabsti* (MNG 10181) housed at the Museum der Natur Gotha, Stiftung Schloß Friedenstein, Germany. B: Complete digital reconstruction in a non-physiological pose. C: Dorsal aspect, D: Ventral aspect. Grey bone models were derived from highest resolution scans (≤ 75μm voxel size); green and turquoise bone models derived from lower resolution scan (≤ 150μm voxel size). Turquoise bone models were slightly re-modelled to account for partially poor visibility of bone within the matrix. Blue bone models were modelled based on superficial visibility from photos and CT-scans and according to detailed description provided by Berman et al. [17]. Shape of thoracic vertebrae was modelled after highly detailed scan of an isolated vertebra (MNG 8966). Further explanation see text.

**Fig 7:**
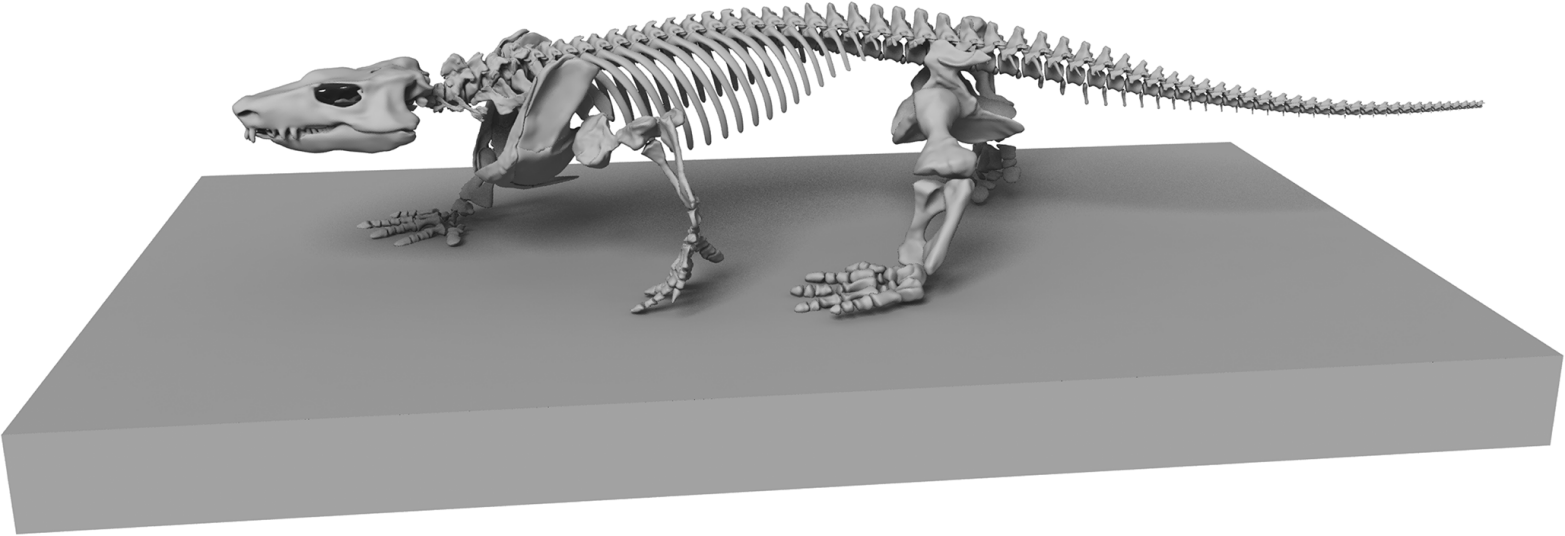
The digital reconstruction of *Orobates pabsti* in a hypothesized naturalistic pose. Stride length, stride width, and manus/pes orientation according to fossil trackways attributed to *Orobates* as the trackmaker [18]. Note that the naturalistic pose presented here requires a suite of assumptions, which should be explored with the help of modelling approaches in future studies. Assumptions include—but are not limited to—the amount of lateral bending of the trunk, the degree of adduction in the proximal limb joints, the relative contribution of pro- and retraction *versus* long axis rotation in the shoulder and hip to stride length. Within the constraints presented by the fossil trackways a range of poses is possible.

The digital skeleton assembled as described above has a total length of 85.14 cm with a snout vent length of 51.09 cm. It consists of “watertight” (i.e., closed surfaces) models of individual bones. The complete digital skeleton of MNG 10181 is presented in an idealized non-physiological pose (Fig 6) as well as in a hypothesized naturalistic pose, which required additional assumptions (Fig 7). The digital skeleton can be scaled to any desirable size. Closed surface bone models can potentially be printed with rapid prototyping techniques and therefore the digital fossil can, for example, be mounted for exhibition purposes. The digital model of MNG 10181 can be found on the data repository of the Stiftung Schloß Friedenstein (http://dx.doi.org/10.17880/digital-reconstruction-of-orobates-pabsti-mng10181) and is available upon request (http://www.stiftungfriedenstein.de/en/contact).

### Body mass

**Fig 8:**
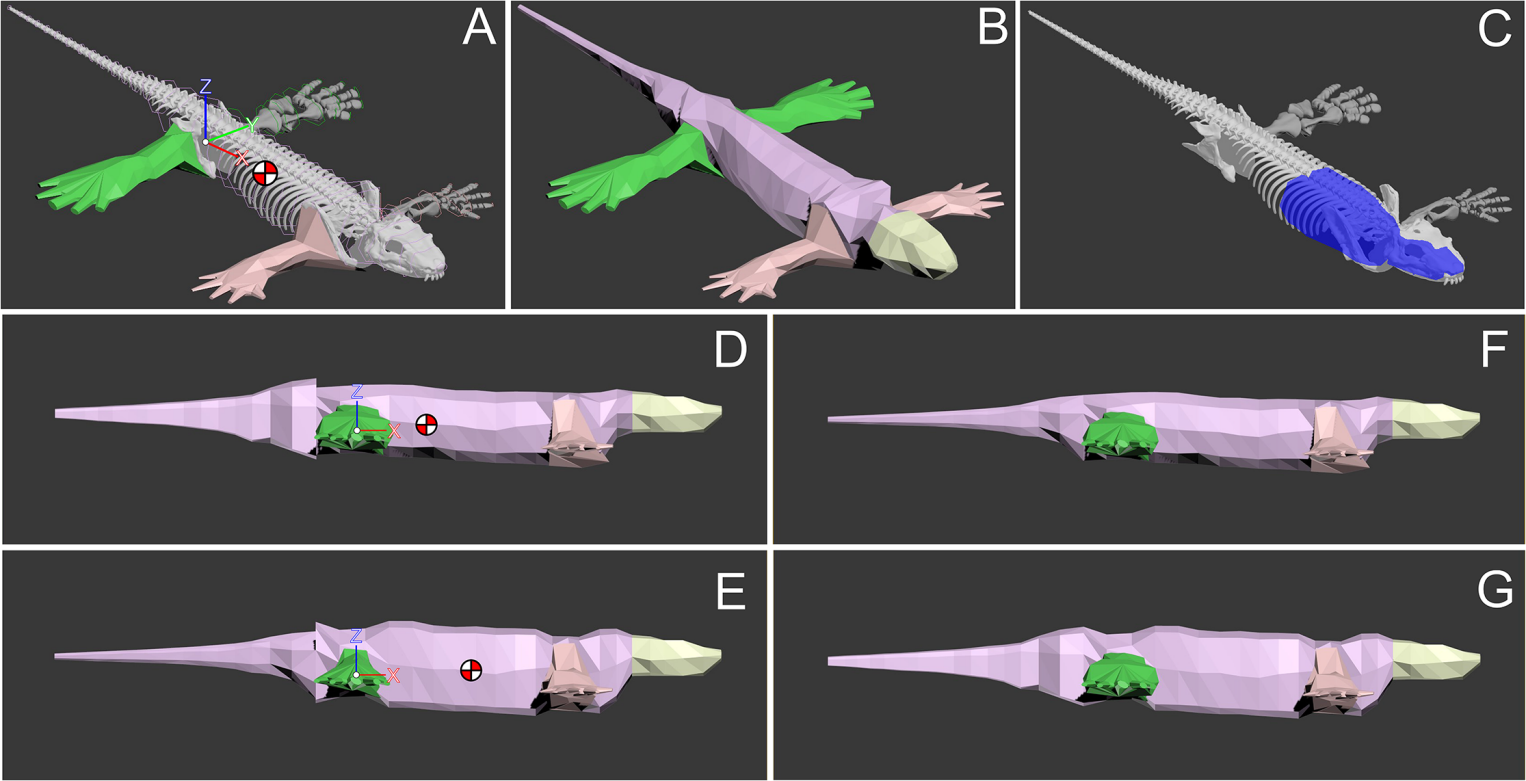
Body mass estimation of MNG 10181. A: Octagonal hugging hoops were used to estimate the body outline. B: Full body outline model. C: Cavities in the oropharyngeal area and lungs were accounted for. D: maximally caudal mass distribution model iteration, with CoM shown (heaviest plausible tail with lightest plausible trunk). E: maximally cranial mass distribution model iteration, with CoM shown (lightest plausible tail with heaviest plausible trunk). F, G: minimal and maximal overall body mass iterations. See text for further explanation.

M_body_ of MNG 10181 was estimated at 3.98 kg in our mean estimate model (Fig 8; mean of minimal and maximal estimated value which are reported in Table 1) with the CoM being located at 12.36 cm cranial to the hips. In both extreme body mass estimations, the position of the CoM relative to the hip differed by approximately one cm. However, when the heaviest tail model was combined with the lightest trunk model and vice versa we found the CoM of M_body_ to range between 8.87 cm and 14.78 cm cranial to the hips. In all cases the CoM was positioned closer to the pelvic girdle than to the pectoral girdle. The head was estimated to have had a mass of 0.21 kg, roughly 5% of the mean estimation model’s M_body_. The neck made up almost 5% of M_body_ (mean estimated value: 0.19 kg). The trunk mass was estimated at 2.05 kg in the mean estimate model (52% M_body_). The tail made up a little more than 9% of M_body_ (mean estimated value: 0.37 kg). The pectoral limb (mean estimated mass: 0.23 kg; 6% of M_body_) of MNG 10181 was considerably lighter than the pelvic limb (mean estimated mass: 0.35 kg; 9% M_body_).

**Table 1:** Whole body and body segment mass estimation. Minimal and maximal mass estimation and position of the respective CoM provided relative to the system origin (*x*, cranio-caudal; *y*, medio-lateral; *z*, dorso-ventral). Max. cranial and Max. caudal represent the extremes of the plausible envelope of the CoM position.

		mass [kg]	*x* [cm]	*y* [cm]	*z* [cm]
Segment					
Head	-	0.213	39.54	-0.05	2.35
Neck	Max.	0.260	32.93	-0.01	1.19
	Min.	0.113	32.93	-0.03	1.82
Trunk	Max.	2.749	13.24	0.01	0.89
	Min.	1.369	11.49	-0.01	1.17
Pectoral limb	Max.	0.273	27.09	-8.93	-1.74
	Min.	0.182	27.22	-8.69	-1.73
Pelvic limb	Max.	0.447	-0.21	-11.15	-0.10
	Min.	0.255	-0.23	-11.48	-0.15
Tail	Max.	0.549	-11.91	0.00	1.71
	Min.	0.182	-11.58	0.01	2.22
Whole					
	Max. cranial	4.460	14.79	0.00	0.59
	Max. caudal	3.502	8.87	0.00	0.72
	Max. overall	5.211	11.79	0.00	0.61
	Min. overall	2.752	12.92	0.00	0.73

### Maximum range of mobility in the shoulder and hip

**Fig 9:**
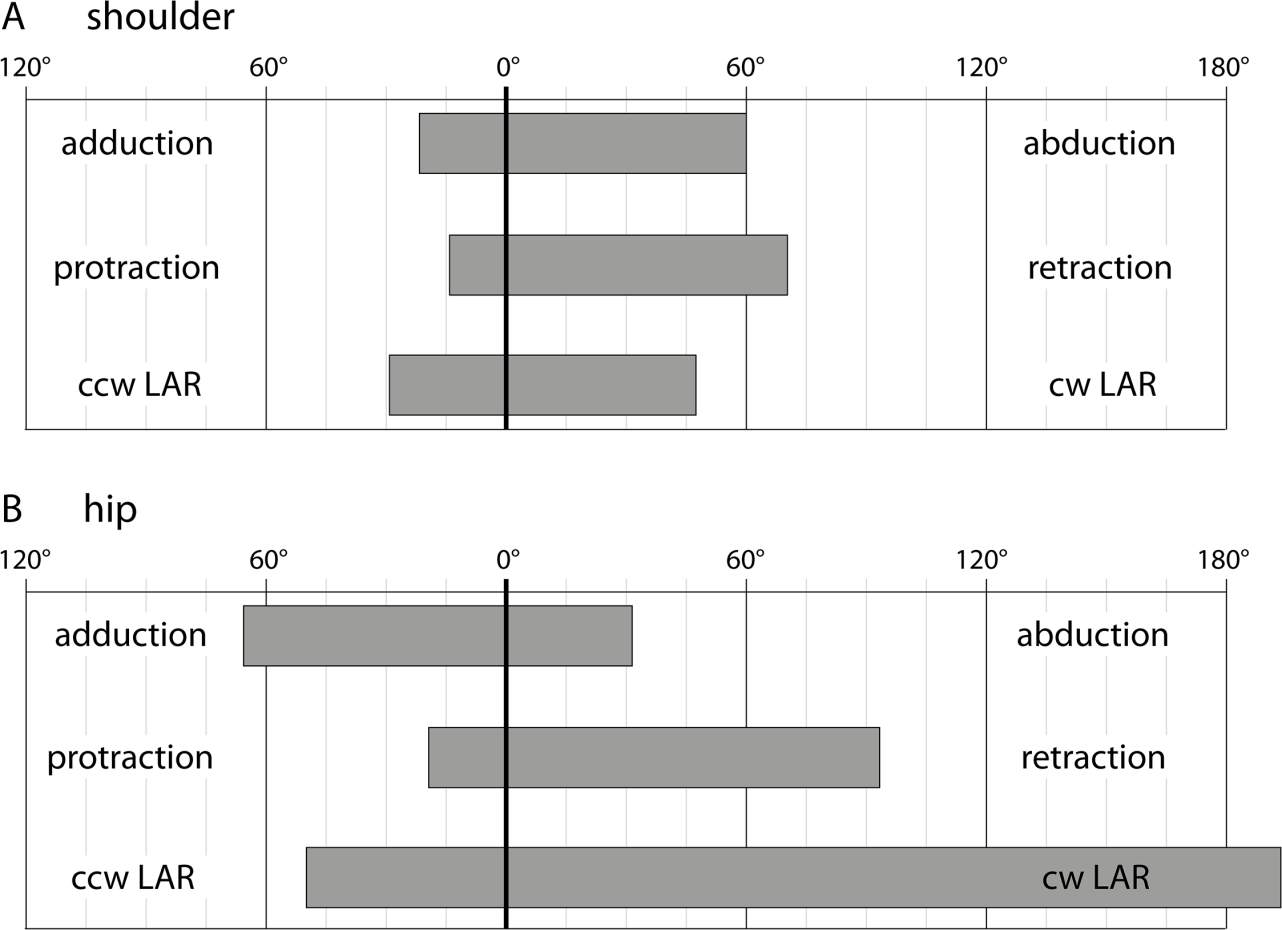
Maximum range of motion in the shoulder (A) and hip (B) joint of *Orobates pabsti* (MNG 10181). All values are relative to the reference pose and were determined from rotation of the stylopodium along axes of the anatomical joint coordinate system until collision of bones with an intermediate joint space of 1.5 mm (further explanation see text).

When assuming an intermediate joint space (1.5 mm) mobility in the shoulder and hip was constrained by bony structures of the glenoid and acetabulum (Fig 9; data for all tested joint spaces see Table 2). In the shoulder, much more abduction than adduction of the humerus was possible until collision of bones. More humeral retraction than protraction, and more counter-clockwise than clockwise LAR (movement of the right humerus when the fossil is viewed from right lateral aspect with the head of MNG 10181 pointing to the right) was possible. In the hip, more femoral adduction than abduction relative the reference pose was possible before bones collided. Femoral protraction was prevented by a bony lip on the acetabulum. Femoral LAR, especially in counter-clockwise direction, was hardly constrained by the acetabulum at all.

**Table 2:** Maximum mobility of the shoulder (humerus) and hip (femur) relative to the reference pose until bone to bone contact in degrees.

	Joint space [mm]	Adduction [°]	Abduction [°]	Protraction [°]	Retraction [°]	LAR cw [°]	LAR ccw [°]
Humerus							
	0	4.01	38.48	3.69	5.49	6.28	41.99
	0.5	9.87	44.84	5.89	17.24	22.70	44.03
	1.0	16.51	52.39	12.5	65.39	26.22	45.13
	1.5	20.79	59.66	14.39	70.68	28.77	46.83
	2.0	28.11	67.50	18.39	72.13	31.16	48.43
	2.5	34.54	70.12	24.09	74.63	35.45	54.77
Femur							
	0	9.12	18.87	9.23	5.72	22.99	32.75
	0.5	22.44	21.97	11.67	86.69	38.32	183.15
	1.0	50.00	26.24	14.74	89.51	42.32	188.73
	1.5	64.96	31.89	18.36	93.05	48.66	194.21
	2.0	70.23	37.52	19.65	96.37	71.14	203.29
	2.5	79.23	42.91	23.65	98.33	123.50	220.72

## Discussion

### A complete digital skeleton of MNG 10181

It is important to point out again that some features were not visible in great detail in our μCT-scans due to thick rock matrix (the thoracic and caudal vertebrae, thoracic ribs) and needed to be (partly) modelled based on other material from the same site and according to previous anatomical description [17]. Nevertheless, the here presented digital reconstruction affords new insights into the postcranial morphology and function of this species.

As noted above, amniotes are a highly successful group of tetrapods that “completed the transition to land” (e.g., [13]) and became independent of aquatic habitats with the development of the amniotic egg. Crown amniotes quickly diversified and exploited terrestrial habitats (e.g., [33]), which likely meant more effective terrestrial locomotion. It remains unknown whether diadectomorphs were reproductively amniote [34], but the analysis of postcranial function on the basis of our complete digital reconstruction of MNG 10181 may help to further characterize its locomotion capabilities and thus provide indirect insight into this pivotal period of the history of tetrapod terrestrial locomotion.

**Fig 10:**
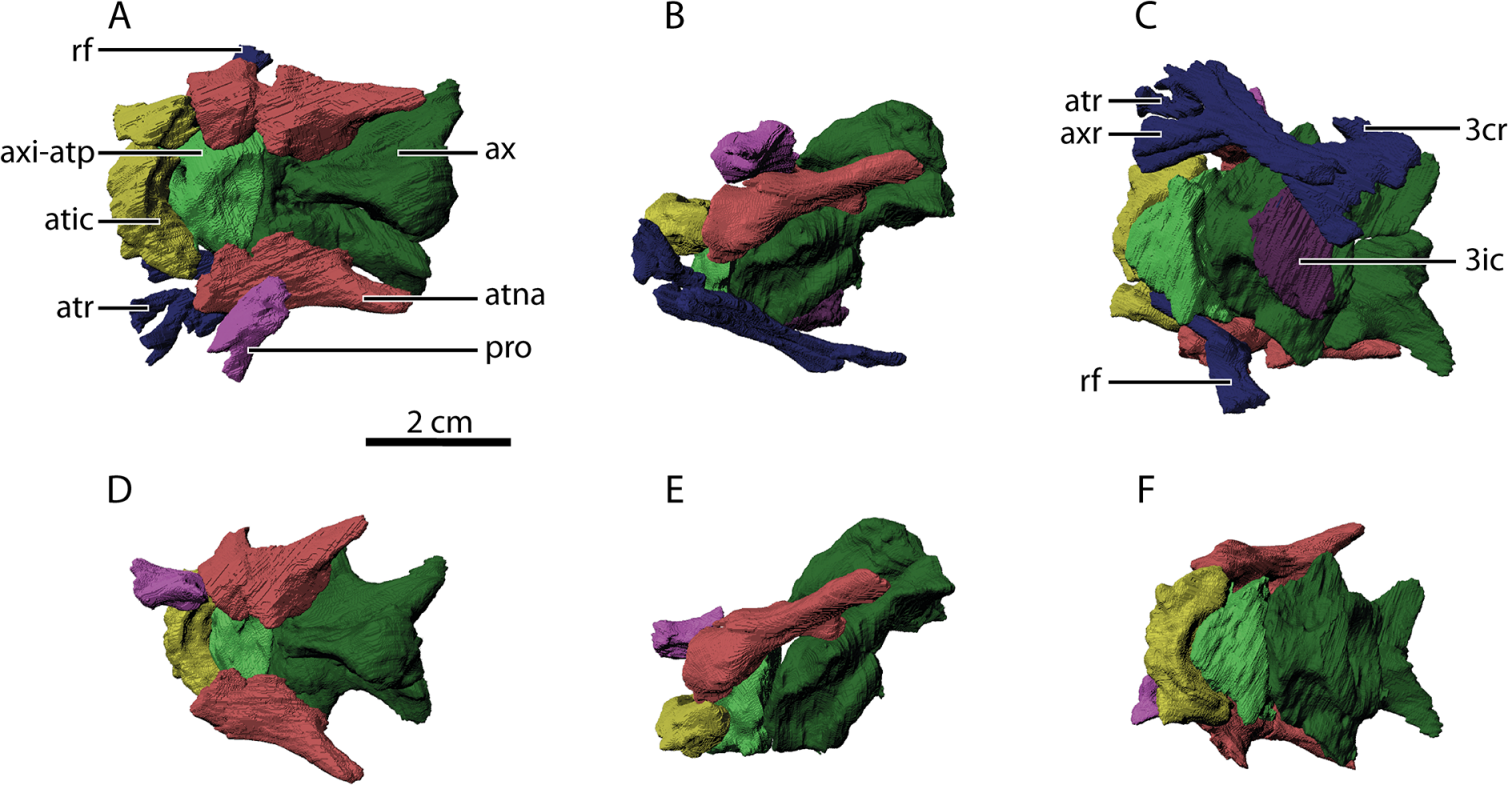
Details of the atlas-axis complex revealed by segmentation of CT image stacks. Anterior directed to the left. A-C: *In situ* location of bone fragments in MNG 10181. D-E: idealized orientation and location of bone fragments (ribs and intercentrum of 3^rd^ cervical not shown). A, D: dorsal aspect, B, E: lateral aspect, C, F: ventral aspect; rf: rib fragment, axi-atp: axial intercentrum plus atlantal pleurocentrum, atic: atlantal intercentrum, atr: atlantal rib, ax: axial neural arch plus axial pleurocentrum, atna: atlantal neural arch, pro: proatlas, axr: axial rib, 3cr: 3^rd^ cervical rib, 3ic: 3^rd^ cervical intercentrum. Please note that all bone models are distorted due to diagenetic processes. Also note the characteristic midventral furrow on the posterior surface of the atlantal intercentrum that receives an anteriorly projecting process of the axial intercentrum also described in *Diadectes* [36].

Our non-destructive examination of the *Orobates pabsti* holotype specimen also revealed previously unknown anatomical detail hidden in surrounding rock matrix. The scan of the cervical vertebral column allowed for detailed digital reconstruction of its anatomical details (Fig 10). The atlas-axis complex is of specific interest as it underwent profound changes during the anamniote to amniote transition (e.g. [35,36]). Evaluation of this complex in MNG 10181 was previously limited to a dorsal view [17]. The new data further substantiates the notion of very similar atlas-axis complexes in both *Diadectes* [35,36] and *Orobates* [17] as well as striking similarities to early crown amniotes (see discussion in [36]). Both, *Diadectes* and *Orobates*, have a midventral furrow on the posterior surface of the atlantal intercentrum that receives an anteriorly projecting process of the axial intercentrum. Our digital models of the comprising structures could potentially be used to test the functional significance of these observed morphological changes (e.g., using musculo-skeletal modelling approaches) across the anamniote to amniote transition in future studies.

### Estimation of mass properties

Estimating body mass properties (here mass and centre of mass, CoM) of an extinct animal is of direct relevance to many areas of palaeobiology. Body mass is a major determinant of both overall metabolic requirements and mass-specific metabolic rate [37–39], and is therefore of direct relevance to analysis of the palaeobiology and palaeoecology of diadectids (food requirements, position in food webs, likely population density etc.).

Body mass (and hence weight) and CoM position are major determinants of the magnitude and orientation (respectively) of the forces an animal exerts against the ground to move. Estimation of these parameters is therefore also a vital first step in inferring the locomotor capabilities of extinct animals (e.g., [28]). However, as CoM position has been shown to undergo considerable ontogenetic change [26,28], the maturity of the analysed specimens can have significant implications for locomotor inference. It is therefore important to note that although our reconstructed specimen of *Orobates* (MNG 10181; skull table length 9.7 cm) appears to have been skeletally mature (based on the overall degree of ossification relative to the apparently more immature specimen MNG 8980; cf. [17]), an appreciably bigger skull (MNG 8760; skull table length 10.7 cm) attributed to this same species has been found, indicating that MNG 10181 may not have achieved maximum size [17].

Previous work on Devonian stem tetrapods such as *Ichthyostega* suggests that they used a belly-dragging gait for terrestrial locomotion, driven mainly by simultaneous forelimb retraction ('crutching' cf. [4,40]). Therefore it seems likely that they exerted greater forces against the ground with their forelimbs than hindlimbs [41]. Later, more terrestrial tetrapods seem to have abandoned this for raised (i.e. non-belly-dragging), predominantly hindlimb-powered, terrestrial locomotion [41,42]. According to our estimation of the whole body CoM position, *Orobates* carried more weight on its hindlimbs. It is therefore reasonable to assume that this species used hindlimb-powered terrestrial locomotion. The absence of belly-drag marks in the trackways assigned to *Orobates* as the trackmaker further suggest that the locomotion was non-belly-dragging [18].

### Mobility in shoulder and hip

Mobility in proximal limb joints is crucial for sprawling locomotion, because of the complex 3D movements that occur here: humeral and femoral abduction/adduction, protraction/retraction, and long axis rotation [43–45]. Hence, range of motion in the shoulder and hip has been quantified in several species that employ sprawling quadrupedal locomotion [31,46]. It is important to distinguish between studies that test for maximum mobility in anesthetized or dead specimens (e.g., [31,46]) from those that analyse the range of motion during *in vivo* locomotion (e.g., [42,45,47–50]). Arnold and colleagues recently demonstrated the large discrepancy between joint range of motion during *in vivo* locomotion and maximum joint mobility in a cadaver [31]. It has also been shown that soft tissues have significant influence on the mobility of a joint, and—since these are in most cases not preserved—inference of mobility based solely on skeletal reconstructions of fossils needs to be very cautious (e.g., [4,31,32,46,51]).

Comparison of mobility in the shoulder and hip of *Ichthyostega* with that of *Orobates* nevertheless indicates fundamental differences in the locomotor systems of these two early tetrapods. Previous work suggests that *Ichthyostega* was not able to make extensive use of its hindlimbs for terrestrial stepping (almost no LAR possible; [4]). In contrast, our data indicate that *Orobates* already had pronounced mobility in the hip joint (similar to that measured in tiger salamanders and Nile crocodiles [4]) with almost no osteological boundaries to LAR. During sprawling locomotion in extant animals, the proximal limb joints engage in a combination of complex 3D movements. This suggests that *Orobates* was at least capable of moving its proximal hindlimb segment within a similarly extensive 3D kinematic envelope to that used by modern sprawlers [44,45], and so may have moved in a similar fashion.

Our results suggest that mobility in the shoulder joint of *Orobates* was more constrained. As in *Ichthyostega* its range of mobility was smaller than that in modern tiger salamanders and Nile crocodiles [4]. In terms of humeral LAR, the maximum mobility in the shoulder of *Orobates* was intermediate between *Ichthyostega* and the modern sprawling species (cf. [4]). Nevertheless, its mobility was likely sufficient to employ similar movements to those recorded during *in vivo* locomotion in the shoulder of alligators [49] and blue tongued skinks [42].

The digital reconstruction presented in this paper will facilitate further biomechanical analysis of the locomotor characteristics of *Orobates* by modelling its locomotion within the constraints provided by the fossil trackways attributed to *Orobates* as the trackmaker. Because of the diadectids’ phylogenetic position close to the crown group node of amniotes, this potentially provides insight into locomotion during the early evolution of amniotes. Moreover, the digital fossil can now be used as an experimental platform to develop hypotheses regarding other behaviours to further narrow down the uncertainty of postcranial musculo-skeletal function of this species. For example, it is likely that *Orobates* and other diadectids dug burrows, mainly using the forelimbs [20,52]. The digital reconstruction can be used to explore matches between potential digging kinematics and characteristics of the burrow morphology.

## Supporting Information

S1 Code Zip-file containing MATLAB code to estimate mass properties and a standard operating procedure (SOP). (ZIP)
