# Supplementary material for: A Three-Dimensional Skeletal Reconstruction of the Stem Amniote Orobates pabsti (Diadectidae): Analyses of Body Mass, Centre of Mass Position, and Joint Mobility
Source: PLoS One. 2015 Sep 10;10(9):e0137284. doi: 10.1371/journal.pone.0137284 (PMC4565719; doi:10.1371/journal.pone.0137284)
Supplement: S1 Code — (ZIP) [file pone.0137284.s001.zip › Mass Properties Code/SOP for mass code.docx]

THINGS TO DO BEFORE YOU START

check that all normals are the right way out (in 3DSmax, select your mesh, go to the ‘modify’ tab [little box on the right dialogue, looks like a blue rainbow], then go to the ‘face’ sub-object level [solid red triangle], and check the ‘show normals’ box. If all of the little blue sticks are not facing outwards on the mesh, click the ‘unify’ button in the ‘surface properties’ box until they all face outwards on the mesh).

check there are no holes (easiest way to do this is in geomagic, where they will show up as green outlines and can be fixed easily with the ‘fill holes’ tool).

TO ESTIMATE MASS PROPERTIES FOR A SINGLE .OBJ FILE

- place the .obj file in the same folder as the mass properties code

- in matlab, use ‘get_body_mass_props.m’ to get mass properties, by typing in the following:

[mass, CoM, tensor_origin, tensor_com] = get_body_mass_props(objname, density, fidelity, units)

Everything in square brackets [] will be outputs, so:

mass = the mass of the object

CoM = the centre of mass of the object

tensor_origin = the inertial tensor for the object about the origin of the coordinate system

tensor_com = the inertial tensor for the object about the objects centre of mass

Everything in normal brackets () are inputs, so:

objname = the name of the .obj file (with ‘’ round it, eg. ‘bone.obj’)

density = the density of the object in kg/m^3^

fidelity = a controller for the amount of parts the object will be split into to estimate its mass properties – 50 is a good value for this

units = the units of the input file, 1 for metres, 0.01 for centimetres, etc. The output will be scaled to metres based on this.

EXAMPLE: [torso_mass, torso_CoM, torso_ITO, torso_ITC] = get_body_mass_props(‘torso.obj’,1060,50,0.01)

This will output four things for an obj file called ‘torso.obj’, using a density of 1060, a fidelity of 50, and units of 0.01 (centimeters)

torso_mass = mass for torso.obj, in kg

torso_CoM = CoM for torso.obj, in m

torso_ITO = inertial tensor about origin for torso.obj

torso_IC = inertial tensor about CoM for torso.obj

TO ESTIMATE MASS PROPERTIES FOR A WHOLE BODY MODEL

- place all .obj files in the same folder as the mass properties code

- make an excel file to tell the code what .obj files to include in the model. This file has one line for each model to be processed. Each line of the excel file needs to be laid out as follows:

Column 1: Classes. These are the groups of mass systems (tails, whole bodies etc). For a single model, you have to put something in here, but it doesn’t matter what. Write ‘whole bodies’ if you don’t want to put anything else.

Column 2: the name of the animal you are modelling (e.g. ‘Pheasant’, ‘Tyrannosaurus’, etc)

Column 3: the name of the iteration of model contained on the line (e.g ‘maximum mass’, etc)

Columns 4 to end: the names of .obj files to be used as mass objects (NB! DO NOT write the ‘.obj’ part on the end of the file name. Anything with the prefix 'MASS_' (e.g. ‘MASS_torso’) will be treated as a solid object. Anything with the prefix ‘CAV_’ (e.g. ‘CAV_lung’) will be treated as a cavity.

- open ‘Batch_Process_Mass_Systems.m’ and scroll down to the ‘USER INPUT’ part. Write the name of your (saved) excel file in the part where it says ‘controller_name’. (NB make sure you put ‘’ round it, e.g. ‘your_controller.xlsx’).

- write the name of your taxa in the part where it says ‘output_name’, again make sure you put ‘’ round it (.e.g ‘your_output’).

- set the density to whatever you want (1060 is normal)

- set the units to whatever you want (remember 1 is meters, 0.01 is centimetres, etc).

- run the script! (green arrow on the top ribbon, or press F5). The script will output an excel file (called ‘your_output.xlsx’ or whatever you put in the ‘output_name’ section) containing the mass and centre of mass for each model detailed in the input excel file.
